# Combination of RCA and DNAzyme for Dual-Signal Isothermal Amplification of Exosome RNA

**DOI:** 10.3390/molecules28145528

**Published:** 2023-07-20

**Authors:** Yuqing Xia, Xin Lei, Xiaochen Ma, Shizheng Wang, Zifu Yang, Yifan Wu, Xiaojun Ren

**Affiliations:** Department of Chemistry and Biology, Faculty of Environment and Life Science, Beijing University of Technology, Beijing 100124, China; xyqing123abc@163.com (Y.X.); lx059593@163.com (X.L.); m13739748941_1@163.com (X.M.); wszwangshizheng@163.com (S.W.); 18684759453@163.com (Z.Y.); 19861900619@163.com (Y.W.)

**Keywords:** nucleic acid analysis, exRNA, signal amplification, DNAzyme

## Abstract

The RNA contained in exosomes plays a crucial role in information transfer between cells in various life activities. The accurate detection of low-abundance exosome RNA (exRNA) is of great significance for cell function studies and the early diagnosis of diseases. However, their intrinsic properties, such as their short length and high sequence homology, represent great challenges for exRNA detection. In this paper, we developed a dual-signal isothermal amplification method based on rolling circle amplification (RCA) coupled with DNAzyme (RCA–DNAzyme). The sensitive detection of low-abundance exRNA, the specific recognition of their targets and the amplification of the detection signal were studied and explored. By designing padlock probes to specifically bind to the target exRNA, while relying on the ligation reaction to enhance recognition, the precise targeting of exosome RNA was realized. The combination of RCA and DNAzyme could achieve a twice-as-large isothermal amplification of the signal compared to RCA alone. This RCA–DNAzyme assay could sensitively detect a target exRNA at a concentration as low as 527 fM and could effectively distinguish the target from other miRNA sequences. In addition, this technology was successfully proven to be effective for the quantitative detection of miR-21 by spike recovery, providing a new research approach for the accurate detection of low-abundance exRNA and the exploration of unknown exRNA functions.

## 1. Introduction

Exosomes originate from endocytic vesicles, and with the formation of early endosomes by plasma membrane invaginations, endosome buds further germinate inward to form multivesicular bodies. Finally, exosomes are secreted by the fusion of the multivesicular bodies and the plasma membrane [1,2]. Exosomes are found in almost all body fluids and are important vectors for RNA, DNA, protein, and lipid transfer, as well as attractive sources of biomarkers for various diseases [3,4,5]. Increasing evidence shows that exosome RNA contains important information related to primordial cells and disease progression, playing an important regulatory role in various pathological processes [6,7,8]. The accurate detection of low-expression exRNA not only contributes to understanding the biological role of exosomes [9], but also provides a new perspective for medical diagnosis [10].

Exosome RNA has the characteristics of low abundance, short sequence, and high sequence homology among family members, which makes it difficult to precisely detect and quantify them [11,12]. Northern blotting is one of the earliest methods for RNA detection, but it can only be used for semi-quantitative detection, having low sensitivity and limitations linked to probe hybridization efficiency [13,14]. Although the RNA sequencing technology has a high detection throughput, data processing is complicated [15,16]. In recent years, microfluidics-based methods have received extensive attention on account of their high integration and precise control of microscale fluids. However, the microfluidics-based technology generally requires separation, labeling and purification of the samples before hybridization and has a high cost [17,18]. Conventional polymerase chain reaction (PCR) requires aa precise temperature control and may produce false positive signals during amplification, which limits the wide application of this method [19,20,21]. In recent years, more and more detection strategies have been reported, including colorimetric methods [22] and electrochemical detection [23,24]. These new methods greatly improve the sensitivity and specificity of RNA detection. Indeed, various isothermal amplification strategies, including loop-mediated isothermal amplification (LAMP) [25], exonuclease-mediated regeneration [26] and polymerization/nicking replication of the analyte [27], have been developed for biosensing. For example, Li et al. performed LAMP to detect the target DNA of *A. baumannii* with the detection limit of 50 pg/μL [28]. However, it is worth mentioning that the LAMP assay requires multiple sets of primers for amplification and is thus prone to background signal enhancement and non-specific amplification [29]. Based on the above reasons, it is crucial to develop an efficient and ultrasensitive method that can accurately detect and quantify exosome RNA.

As one of the most widely used isothermal amplification methods, RCA allows for the mass production of periodic long, single-strand DNA with predesigned sequences [30,31]. Typically, the reaction is initiated by the hybridization of the target and a padlock probe. Following hybridization, a ligase is added into the system to close the padlock probe into a loop and form a circular template. With the help of primers, the short-stranded nucleic acid expands continuously after 2′-deoxynucleotide-5′-triphosphate (dNTPs) and DNA polymerase are added, leading to signal amplification. Signal amplification using RCA can, in theory, amplify the signal 10^9^ times. In addition, padlock probe-based RCA can target short RNAs and discriminate highly similar sequences to genotype RNAs [32]. Combining RCA with other techniques allows for better signal amplification. Zhuang et al. pioneered the combined use of RCA and CHA to form deoxyribozymes for the detection of miRNAs [33]. Later, Luo’s group developed a highly specific nucleic acid detection platform for the simultaneous quantification of several extracellular vesicles (EV)-derived miRNAs at a constant temperature by integrating the advantages of a clustered regularly interspaced short palindromic repeats/CRISPR-associated nucleases (CRISPR/Cas) system and RCA [34]. In conclusion, RCA has the advantages of rapid amplification and strong specificity, which make it a promising agent in the fields of RNA detection and biomedicine.

DNAzyme consists of single-stranded DNA fragments synthesized by in vitro molecular evolution, which has the function of protease (such as horseradish peroxidase) or endonuclease [35]. Typically, DNAzyme consists of a catalytic core region and two substrate-binding arms that bind to the substrate through complementary base pairing and can be activated, serving as enzymes, upon binding with specific metal ions [36]. When DNAzyme specifically binds to metal ions (including Pb^2+^, Ca^2+^, Na^+^, UO_2_^2+^ and Mg^2+^), it can efficiently cleave the phosphodiester bonds of the RNA base rA in substrate probes [37] sequentially, outputting response signals via labeled substrate probes. For example, Zhang et al. proposed a label-free, dually amplified and homogeneous DNAzyme assay for the sensitive detection of lead pollution [38]. Wang’s group developed an amplifier consisting of a modularized DNAzyme-amplified two-stage-cascaded hybridization chain reaction (CHCR−DNAzyme) circuit. This Mg^2+^-dependent autonomous nonlinear enzyme-free signal amplification paradigm identified EV through a highly sensitive and selective detection of their inherent miRNAs in situ [39]. DNAzymes have many advantages: (1) a DNAzyme sensing system has good selectivity and flexibility, and the sequences can be designed independently to catalyze specific units; (2) the traditional RNases are susceptible to the temperature and may become deactivated at high or low temperatures, while DNAzyme is not affected by the temperature and has higher stability; (3) the synthesis of DNAzyme is relatively simple and low-cost. For these reasons, DNAzyme has the potential to be an ideal candidate for developing amplification sensing platforms for detecting RNA [40], DNA [41] and proteins [42].

**Scheme 1:**
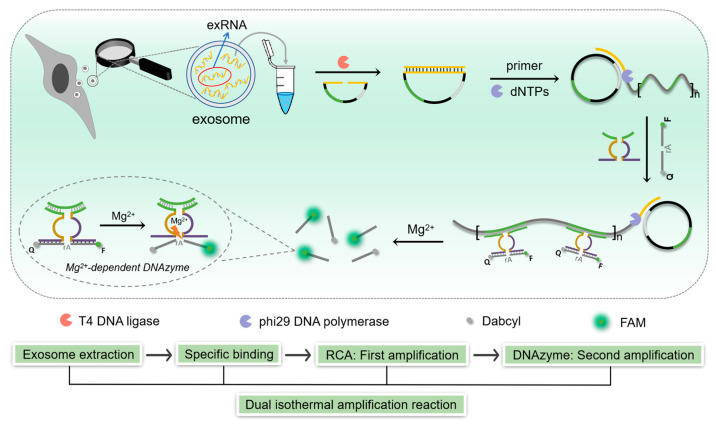
Schematic illustration of the dual-signal isothermal amplification strategy based on RCA coupled with the DNAzyme (RCA–DNAzyme) assay for exRNA analysis.

Here, we propose a dual-signal isothermal amplification technology based on the rolling circle amplification reaction coupled with DNAzyme, with the combination of the high amplification efficiency of the RCA reaction and the characteristics of the DNAzyme autocatalytic cleavage substrate to release fluorescence (Scheme 1). Inspired by the functionalization potential of the long programmable strands produced by RCA, we hypothesized that these scaffolds could be loaded with functional groups precisely. Through the design of a template sequence, the scaffold was programmed to precisely hybridize with DNAzyme for an optimal response. Driven by exRNA, the target-induced RCA began to work. Furthermore, the DNAzyme cleavage reaction induced by Mg^2+^ enabled the detection and analysis of exRNA via fluorescence recovery. The RCA–DNAzyme assay has the advantages of strong specificity, high sensitivity, and constant reaction temperature. This dual-signal isothermal amplification strategy is expected to provide new technical support for the accurate detection of low-abundance exRNA and the early diagnosis of diseases.

## 2. Results and Discussion

### 2.1. Design and Working Principle of the RCA–DNAzyme Assay

The principle of the RCA–DNAzyme assay is illustrated in Scheme 1. In this strategy, the recognition region of the padlock probe was designed to precisely target the unique sequence of exRNA. Upon hybridization of exRNA with the phosphorylated padlock probe, the 3′- and 5′-termini of the padlock probe were brought into proximity and then were ligated to produce a cyclized probe under the catalysis of the T4 DNA ligase. Then, the RCA reaction was triggered under the action of Phi29 DNA polymerase and using a short DNA primer, achieving the first signal amplification. The produced linear repetitive RCA amplicon with labeled modules recruited the DNAzyme structure by specific hybridization. The hybridization between DNAzyme and the RCA products activated the cutting activity of Mg^2+^, which further autocatalyzed the cleavage of the DNAzyme substrates (labeled with FAM and dabcyl at the 5′ and 3′ ends, respectively). Then, the fluorophores were released and realized the second signal amplification. The design of the dual-signal amplification strategy is advantageous for the sensitive detection of-low abundance exRNA. It is worth noting that the DNAzyme signal amplification process can be performed under the condition of constant temperature and without an enzyme, which is a significant cost saving.

### 2.2. Feasibility Validation of the RCA–DNAzyme Assay

**Figure 1:**
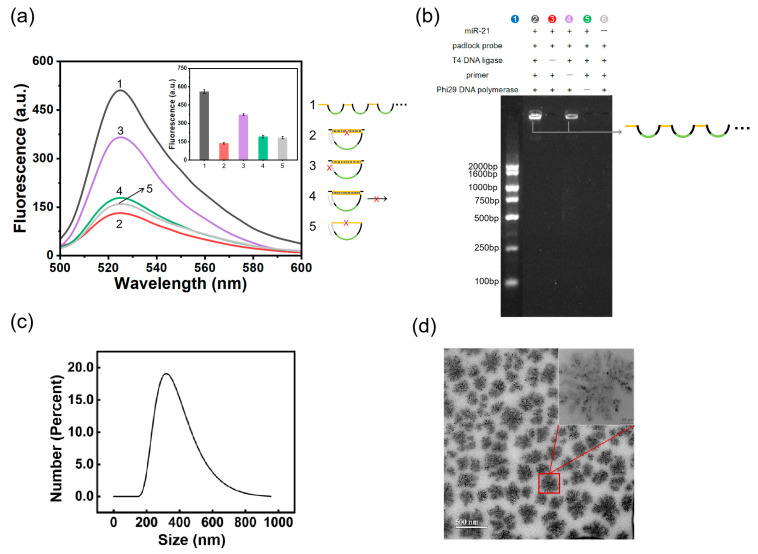
Mechanism verification of the RCA–DNAzyme assay. (**a**) Fluorescence spectrogram of the exRNA-induced reaction: ① experimental group, ② no T4 DNA ligase, ③ no primer, ④ no phi29 DNA polymerase and ⑤ blank control (no target); (inset) the fluorescence intensity of miR-21 was determined at the 525 nm excitation wavelength. (**b**) Electrophoretic analysis of the miR-21-triggered RCA process. Lane 1 shows the DNA marker. (**c**,**d**) Characterizations of the RCA products. Particle size distribution of the RCA products by DLS (**c**) and TEM images of the RCA products (**d**). Scale bar: 500 nm.

In order to verify the feasibility of the dual-signal isothermal amplification technology based on RCA coupled with DNAzyme, we first performed the assay to amplify a synthesized sequence of miR-21. Figure 1a illustrates that the experimental group showed an obvious strong fluorescence signal, whereas almost no signal was observed when no padlock probe was used. Particularly, it can be seen that neither the absence of T4 DNA ligase nor that of phi29 DNA polymerase significantly enhanced the fluorescence, indicating the necessity of the ligation process and the amplification process for the detection of exRNA. However, when the primers were absent, partial fluorescence signals were produced, possibly because of the strong polymerizing activity of phi29 polymerase, which promoted the generation of the RCA products. The performance (feasibility, sensitivity and specificity) of the DNAzyme structure was also validated, and the results are presented in Figures S1, S3 and S4.

An agarose gel electrophoresis analysis further demonstrated the RCA process (Figure 1b). We detected a large amount of products with a high molecular weight located in the sampling well in the presence of miR-21 (Lane 2), confirming the generation of RCA products. The band was very clear, which is in sharp contrast to the lack of signal in the blank control (Lane 6). Meanwhile, the RCA products were dramatically reduced when primer deletion occurred, possibly because primer deletion affected the efficiency of the RCA reaction (Lane 4). When the T4 DNA ligase or phi29 DNA polymerase was absent, no RCA products were generated (Lane 3 and Lane 5), indicating that both T4 DNA ligase and phi29 DNA polymerase are indispensable in the reaction process. At the initial stage of the reaction, the RCA products first formed an amorphous structure; with the progress of the reaction, crystalloid structures on the matrix’s surface appeared. Subsequently, the surface of these spherical structures continuously assembled to form a petal structure and, gradually, a flower-like structure. The characterization of the RCA products by dynamic light scattering (DLS) and transmission electron microscopy (TEM) is presented in Figure 1c,d. It was revealed that the RCA products were snowflake-like, nearly round, monodisperse nanoparticles with a uniform diameter of about 350 nm.

### 2.3. Optimization of the Experimental Conditions

**Figure 2:**
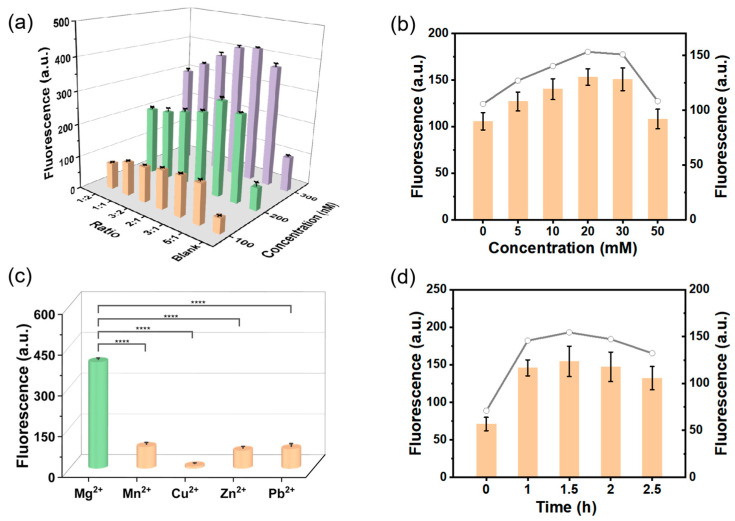
Effect of the DNAzyme substrate and other conditions on the fluorescence signal intensity. (**a**) Three-dimensional histogram of the relationship of different proportions of substrate concentrations and DNAzyme structure with fluorescence signal intensity, when the substrate was 100 nM, 200 nM and 300 nM. (**b**) The relationship between the concentration of Mg^2+^ and fluorescence signal intensity. (**c**) Fluorescence signal responses of DNAzyme fueled by different ions; concentration: 20 mM. (Two-tailed Student’s *t* test; bars represent mean ± SD; **** *p* < 0.0001. (**d**) Relationship between cutting time of DNAzyme structure and fluorescence signal intensity.

In order to achieve the best detection performance of the dual-signal amplification strategy, several conditions were optimized using the synthetic DNAzyme open sequence. The DNAzyme open sequence is the partial sequence of the RCA products that can be hybridized with the DNAzyme structure. It is commercially available and was used to verify the feasibility of the DNAzyme structure. The effects of ration between DNAzyme structure ratio and substrate concentration (DNAzyme/substrate) on the signal amplification efficiency were first investigated. As shown in Figure 2a and Figure S2, the best fluorescence signal was recorded when the DNAzyme structure ratio was 3:1. At this ratio, we found that the signal-to-noise ratio was the highest when the substrate concentration was 200 nM. The concentration of Mg^2+^ also influences the cleave efficiency of DNAzyme. The fluorescence signal intensity reached the peak when Mg^2+^ concentration was 20 mM (Figure 2b). We also confirmed that other metal ions, such as Mn^2+^, Cu^2+^, Zn^2+^ and Pb^2+^, were unable to fuel DNAzyme to cleave substrates, as shown in Figure 2c, laying the groundwork for the specific detection of exRNA. In addition, the cleavage time of the DNAzyme substrate is also an important factor affecting the fluorescence signal. It can be seen that the signal-to-noise ratio was the highest when DNAzyme was incubated for 1.5 h (Figure 2d).

### 2.4. Quantification Performance of the RCA–DNAzyme Assay

**Figure 3:**
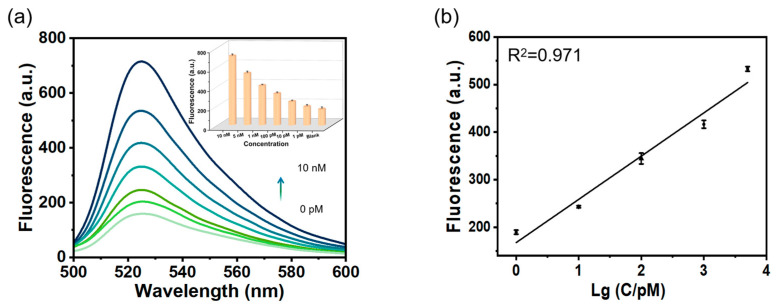
Sensitivity analysis of miR-21 based on the RCA–DNAzyme assay. (**a**) Typical fluorescence spectra obtained with different concentrations of miR-21, i.e., 0, 1 pM, 10 pM, 100 pM, 1 nM, 5 nM, 10 nM (from bottom to top); (inset) the fluorescence intensity of miR-21 was determined at the 525 nm excitation wavelength. (**b**) The linear relationship between the logarithmic (lg) value of miR-21 concentration and fluorescence intensity. The error bars indicate the standard deviations obtained with three individual measurements (*n* = 3).

Under the optimized experimental conditions, different concentrations of miR-21 were analyzed to investigate the sensitivity of the proposed assay. The relevant fluorescence spectra are shown in Figure 3a, which represents the relationship between the concentrations of miR-21 and the fluorescence intensities at 525 nm. The logarithmic (lg) value of miRNA concentration and fluorescence intensity showed a good linear relationship in the concentration range of 1 pM~10 nM (Figure 3b). The correlation regression equation was fitted to Y = 95.168X + 159.129, and the correlation coefficient was 0.971. Considering three times the standard error over the blank response, the detection concentration limit was 527 fM. The sensitivity was higher than that of most amplification-based fluorescence methods reported previously (Table S3) [43,44,45,46].

### 2.5. Specificity Evaluation of the Assay

**Figure 4:**
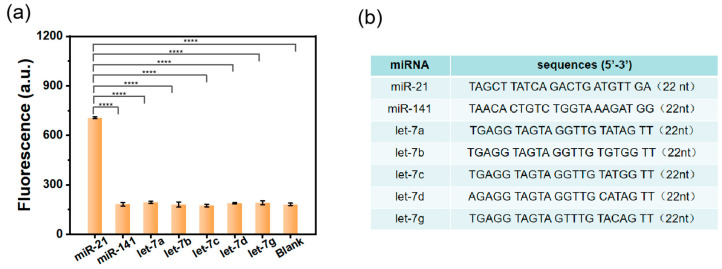
Fluorescence response of the RCA–DNAzyme assay towards different miRNAs t a concentration of 10 nM. (**a**) Fluorescence intensity values measured for different miRNA at the 525 nm emission wavelength. Statistically significant data were obtained by the two-tailed unpaired Student’s *t*-test: **** *p* < 0.0001. (**b**) The sequences used in this work for exRNA specific detection.

ExRNA such as miRNA sequences are short and highly similar; so, it is still a big challenge to specifically distinguish different miRNAs. In order to study the specificity of the proposed detection method, different miRNA sequences at the same concentration were analyzed under the same experimental conditions. The specificity test for miR-21 was carried out by challenging the system with other sequences, including miR-141 and the let-7a family. As can be seen in Figure 4, the fluorescence signal of the target miR-21 was the strongest, and the fluorescence signal intensity of other different miRNA sequences was equivalent to that of the control group.

### 2.6. Characterization of Exosomes and Detection of miR-21

**Figure 5:**
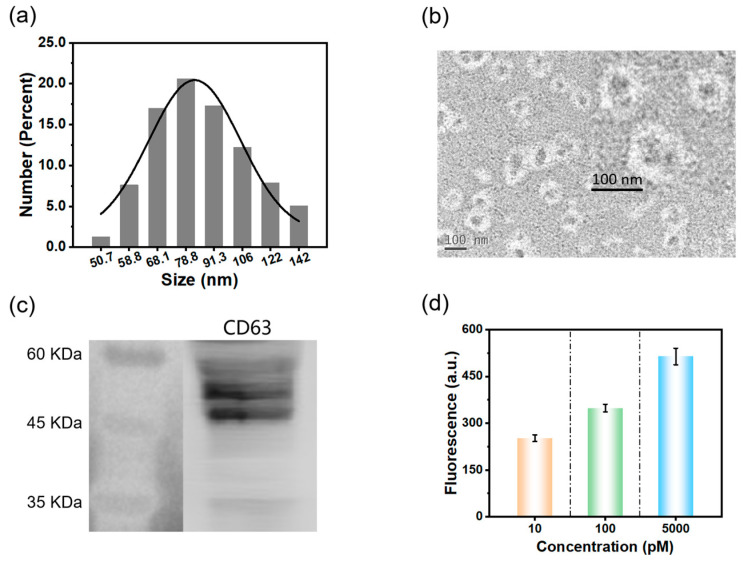
Characterization of exosomes secreted by HeLa cells and detection of miR-21 by spike recovery. (**a**) Particle size distribution diagram of exosomes obtained with DLS and Gauss distribution curve. (**b**) TEM image of exosomes. (**c**) Western blot analysis of exosomes’ characteristic proteins. (**d**) Fluorescence intensities of the spike recovery detection of miR-21 at different concentrations (10 pM, 100 pM and 5000 pM) in cell supernatants.

We first characterized the diameter, morphology and characteristic proteins of Hela exosomes. The size and distribution were measured and analyzed by a dynamic light scattering particle size analyzer (Figure 5a). It can be seen that the extracted exosomes dispersed evenly, and their particle size was mostly concentrated in the range of 68.1~91.3 nm, with an average particle size of 80.4 ± 2.6 nm. The morphological characteristics of the exosomes were observed by TEM. As shown in Figure 5c, HeLa exosomes are membranous disc-like structures, about 80 nm in diameter. The expression of the characteristic protein CD63 in the exosomes was further confirmed by Western blot. In Figure 5d, CD63 appears as a continuous band with a size of 37~50 KDa. The above results indicated that the exosomes were successfully extracted and had regular morphology and uniform particle size.

Subsequently, different concentrations of miR-21 were added to the 10-fold diluted cell supernatant for spiked recovery to evaluate the accuracy of the RCA–DNAzyme assay. As shown in Figure 5d, for targets with a concentration in the dynamic range of 10~5000 pM, the recovery was in the range from 95.10% to 107.41%. Moreover, the relative standard deviation (RSD) was below 5.18% (Table 1), indicating the acceptable accuracy of the sensing strategy. We used DEPC as a baseline negative control and tested the fluorescence emission spectra of DEPC and a blank control before the experiment. The detection results in the cell supernatants and of DEPC are presented in Figure S5. We also evaluated the spiked recovery capability of this method for DEPC (Figure S6). The results demonstrated that the proposed RCA–DNAzyme assay could be considered as a robust and reliable method to detect exRNA in complex biological samples.

## 3. Materials and Methods

### 3.1. Materials and Reagents

The DNA sequences were provided by Ruibo Xing Ke Biotechnology Co., Ltd. (Beijing, China), and fluorophore-modified DNAzyme was purchased from Sangon Biotechnology Co., Ltd. (Shanghai, China). All sequences used in the experimentsa were purified by HPLC and are listed in Table S1 (Supporting Information). Adenosine triphosphate (ATP), T4 polynucleotide kinase (T4 PNK), T4 DNA ligase, Phi29 DNA polymerase, the deoxyribonucleotide triphosphates mix (dNTPs) and the RiboLock Rnase inhibitor were bought from Thermo Fisher Scientific (Shanghai, China) Co., Ltd. Diethylpyrocarbonate (DEPC)-treated water, the nucleic acid dye Gel-Red, the DNA loading buffer and the tris-acetate-EDTA (TAE) electrophoresis buffer were obtained from Beyotime Biotechnology Co., Ltd. Agarose was provided by Beijing Shengke Yusheng Technology Co., Ltd. (Beijing China), the 100 bp DNA marker was purchased from Beijing Bailingwei Technology Co., Ltd. (Beijing, China).

HeLa (human cervical carcinoma cell line) cells were provided by the Union Cell Bank of the Chinese Academy of Medical Sciences. Dulbecco’s Modified Eagle Medium (DMEM, high glucose) and phosphate-buffered solution (PBS) were obtained from Hyclone (Logan, UT, USA). Fetal bovine serum (FBS) and 0.25% trypsin were purchased from Gibco BRL (Carlsbad, CA, USA). The serum-free cell cryopreservation solution was provided by New Cell & Molecular Biotechnology Co., Ltd. All experiments were performed using DEPC-treated water to minimize the effect of RNase on exRNA detection.

### 3.2. Detection of exRNA Using the RCA–DNAzyme Assay

The detection of the dual-signal amplification was divided into two parts: the RCA reaction and the cutting of the DNAzyme structure. We mixed 2 μL of padlock probe (10 μM), 2 μL of ATP (10 mM), 2 μL of 10 × T4 PNK buffer and 1 μL of T4 PNK (10 U/μL) and incubated the mixture at 37 °C for 1 h to prepare a phosphorylated padlock probe. The ligation step was performed at 25 °C for 1 h in a volume of 20 μL, including 1 μL of phosphorylated padlock probe (400 nM), 1 μL of exRNA, 2 μL of 10× T4 DNA ligase buffer and 1 μL of T4 DNA ligase (5 U/μL). Then, 2 μL of primer (500 nM), 4 μL of dNTPs (10 mM), 3 μL of phi29 DNA polymerase buffer and 1 μL of phi29 DNA polymerase (10 U/μL) were added to ensure the smooth operation of the RCA reaction. The mixture was reacted at 37 °C for 1 h and then heated to 80 °C for 10 min to terminate the RCA process. The cutting of the DNAzyme structure was carried out by mixing HEPES buffer (pH = 7.4, 0.5 M), DNAzyme 1 (3 μL, 10 μM), DNAzyme 2 (3 μL, 10 μM), substrate (1 μL, 10 μM), MgCl_2_ (1 μL, 1 M) and NaCl (5 μL,1 M). The RCA products were added to the above mixture and incubated at 25 °C for 1.5 h to ensure the sufficient cleavage of the substrate.

### 3.3. Gel Electrophoresis Analysis and Fluorescence Measurements

After mixing 5 μL of the RCA products and 1 μL of loading buffer, the sample was loaded into the loading well of a freshly prepared 2% agarose gel. Using a DNA Marker as the control, electrophoresis was performed at the fixed voltage of 160 V for 25 min in 1× TAE buffer (40 mM Tris-acetic acid, pH 8.5, 2 mM EDTA). Imaging was performed using a Tanon 5200 Multi system.

The fluorescence signals were recorded using the molecular device microplate reader spectraMax M4, Molecular Devices, (San Jose, California, USA) at room temperature. The excitation wavelength for fluorescence detection was 488 nm, with a step size of 5 nm and a scanning range of 500~600 nm.

### 3.4. Dynamic Light Scattering and Transmission Electron Microscopy

The size and morphology of the RCA products were further characterized by DLS and TEM. In total, 50 µL of the RCA products was diluted to 1 mL and added into the sample tank of the particle size analyzer. The particle size of the RCA products was measured by the dynamic light scattering particle size analyzer. The morphology of the RCA products was observed by TEM as follows. We placed a drop of 10 µL of the RCA product solution onto a copper mesh and let it stand for 30 min to dry. Subsequently, the morphology of the RCA products was observed by TEM.

### 3.5. Cell Culture and Exosomes Extraction

HeLa cells were incubated in DMEM medium containing 10% FBS in cell culture flasks at 37 °C in a humidified atmosphere with 5% CO_2_. The exosomes were extracted by ultracentrifugation. When the number of cells in the flask grew to 70%~80% saturation, the medium was removed, and the cells were washed three times with PBS and cultured in serum-free medium for 24~48 h. Then, the medium was collected (supernatant). First, the supernatant was centrifuged at 300 g/min for 10 min to remove the cells in the sample. Then, it was carefully transferred to a new centrifuge tube and centrifuged at 2000 g/min for 20 min to remove the dead cells. Finally, it was transferred to a new centrifuge tube and centrifuged at 10,000 g/min for 30 min to remove the cell debris. All centrifugation steps were performed at 4 °C. The resulting supernatant was filtered with a 0.22 μm filter membrane to remove larger particles such as extracellular vesicles, and then centrifuged at 100,000 g/min for 70 min using an ultracentrifuge SW32-Ti horizontal rotor. The obtained precipitate was resuspended in PBS and centrifuged at the same speed for 80 min. The obtained precipitate consisted of the exosomes of HeLa cells. Finally, the above precipitate was resuspended in 100 μL of PBS. The obtained exosomes could be stored in a refrigerator at −80 °C for a long time.

### 3.6. Characterization of Exosomes

The diameter and distribution of HeLa exosomes was measured by DLS, as follows. Take 100 µL exosomes suspension and dilute it to 1 mL, then add it to the sample tank of the particle size analyzer. The particle size of the exosomes was measured using the dynamic light scattering particle size analyzer.

The morphology of the HeLa exosomes was observed by TEM. The preparation process was as follows. The isolated exosomes were resuspended in PBS at an appropriate concentration, and a drop of solution (about 10 µL) was added to the copper grid for 2 min and then dried with a filter paper. A drop of 3% phosphotungstic acid (pH 7.0) was used for negative staining for 2 min. Finally, we washed the mesh with a drop of double-steaming water, then blot it with filter paper and let it rest for 30 min to dry.

Western blot was used to detect the expression of exosomes’ signature proteins. First, total proteins from exosomes were extracted using the ProteinExt^®^ Mammalian Nuclear and Cytoplasmic Protein Extraction Kit. The concentration of the proteins was measured by the Protein Quantification Kit. Finally, polyacrylamide gel electrophoresis was performed, and the target protein bands were detected by a gel imaging system.

## 4. Conclusions

In this work, a dual-signal isothermal amplification technique based on rolling circle amplification coupled with DNAzyme was designed, which achieved the precise targeting and sensitive detection of low-abundance exRNA. The precise targeting of exosome RNA was achieved by designing a padlock probe, at the same time relying on the combined reaction to enhance the recognition mechanism. Furthermore, the dual-signal amplification strategy significantly enhanced the fluorescence signal. The reaction was performed under constant temperature, which avoided the use of temperature control instruments and allowed for better reaction stability. The RCA–DNAzyme strategy can be used to detect the target exRNA at a concentration as low as 527 fM. Finally, the dual-signal isothermal amplification strategy was applied to the detection of the spiked target miR-21 in cell samples, and the recovery results demonstrated the high accuracy of the RCA–DNAzyme assay. Considering its sensitivity and simplicity, this assay provides a new analytical tool for the precise detection of low-abundance exRNA, functional studies of exosome RNA, and the early diagnosis of diseases.

## Figures and Tables

**Table 1:** The results of spike recovery in cell supernatants.

Samples	Spiked (pM)	Detected (pM)	Recovery (%)	RSD (%)
1	10	9.51	95.10	4.25
2	100	98.17	98.17	3.55
3	5000	5370.32	107.41	5.18

Relative standard deviation (RSD) was obtained by three parallel experiments.

## Data Availability

Not applicable.

## Supplementary Materials

The following supporting information can be downloaded at: https://www.mdpi.com/article/10.3390/molecules28145528/s1, Figure S1: Verification of the feasibility of the DNAzyme structure; Figure S2: Optimization of the structure concentration and the ratio of DNAzyme; Figure S3: Fluorescence emission spectra after the addition of DNAzyme open sequences at different concentrations (from bottom to top): 0, 1 nM, 5 nM, 10 nM, 50 nM and 100 nM; Figure S4: Specificity assessment of the DNAzyme structure; Figure S5: Fluorescence emission spectra of the baseline negative control and blank control in cell supernatants (a) and DEPC (b); Figure S6: Fluorescence intensity of miR-21 spike recovery detection at different concentrations (10 pM, 100 pM and 5000 pM) in DEPC. Table S1: The sequences and their secondary structure prediction used in the RCA–DNAzyme assay; Table S2: Nucleic acid sequences used in the specificity evaluation of the DNAzyme structure; Table S3: The results of spike recovery in DEPC; Table S4: Comparison of different methods for RNA detection.
