# Supplementary material for: Combination of RCA and DNAzyme for Dual-Signal Isothermal Amplification of Exosome RNA
Source: Molecules. 2023 Jul 20;28(14):5528. doi: 10.3390/molecules28145528 (PMC10384651; doi:10.3390/molecules28145528)
Supplement: Supplementary file 1 [file molecules-28-05528-s001.zip › molecules-2411511-supplementary.pdf]

Supporting Information

Collision of RCA and DNAzyme for dual-signal isothermal amplification of exosome RNA

Yuqing Xia [1a], Xin Lei [1b], Xiaochen Ma [1], Shizheng Wang [1], Zifu Yang [1], Yifan Wu [1], and Xiaojun Ren [1,*]

[1] Department of Chemistry and Biology, Faculty of Environment and Life Science, Beijing University of Technology, Beijing 100124, China
* Correspondence: xjren@bjut.edu.cn

Design of sequences

The padlock probe includes a target recognition module, a primer hybridization module and a label module. The target recognition module specifically binds the target exRNA based on the principle of base complementary pairing, thereby achieving precise targeting of exRNA. With the help of short DNA primer and phi29 DNA polymerase, the primer hybridization module can initiate the RCA reaction to achieve first signal amplification. The RCA products of the tag module can hybridize with the DNAzyme structure to form a special spatial structure, and then autocatalyze the ribonucleic acid substrate under the action of Mg^{2+}, realizing the second amplification of the signal with the releasing of fluorophore. MiR-21, padlock probe, primer, DNAzyme1, DNAzyme2, substrate and their secondary structure prediction results are shown in Table S1.

Table S1. The sequences and their secondary structure prediction used in the RCA-DNAzyme assay.

Name	Secondary structure	Sequences（5′- 3′）	Free energy（kcal/mol）
miR-21		TAGCT TATCA GACTG ATGTT GA (22 nt)	0
padlock probe	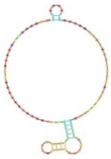	CTGAT AAGCT AAACT ATACA ACATA CTACC TCACT CCTTG TTGAC CCTGG AGGGT AGGAG GTGCA TTCAT ATTCG TCGTC AACAT CAGT (89 nt)	-6.53
primer		TGAGG TAGTA TGTTG TATAG TT (22 nt)	0
DNAzyme1	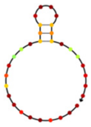	CTCCT TGTTG ACGTT ACACC CATGT TACTC TC (32 nt）	-0.94
DNAzyme2	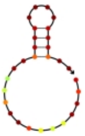	TGATA TCAGC GATTA ACCCT GGAGG GTAG (29 nt）	-3.56
substrate		GAGAG TAT(rA)G GATAT CA (17 nt）	0

Note: The secondary structure and free energy of the designed sequences were predicted by NUPACK (http://www.nupack.org) to minimize the influence of sequence spatial structure on the experimental results.

DNAzyme structure verification

The DNAzyme-open sequence (CTACC CTCCA GGGTC AACAA GGAG) was synthesized to verify that the DNAzyme structure can couple with RCA products to turn on fluorescence. Five parallel experiments were carried out as control. According to Figure S1, the DNAzyme structure has the ability to detect targets sensitively. When any sequence in the DNAzyme structure is missing, the fluorescence signal intensity is much lower than that of the experimental group, indicating that the probe sequence is indispensable.

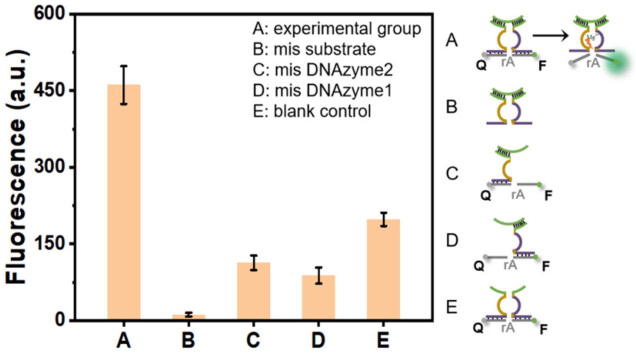

Figure S1. Verification of the feasibility of DNAzyme structure. (A) experimental group. (B) mis substrate. (C) mis DNAzyme2. (D) mis DNAzyme1. (E) blank control (no DNAzyme-open).

Optimization of reaction conditions

The concentrations of substrate and the ratio of DNAzyme structure have great influence on fluorescence signal intensity. In order to explore the best experiment conditions, six parallel experiments were carried out. The ratio of DNAzyme to the substrate was 1:2, 1:1, 3:2, 2:1, 3:1, 5:1, respectively. It can be seen from Figure S2 that the intensity of the fluorescence signal gradually increases as the ratio of DNAzyme to substrate increases. When the structure ratio is 3:1, the intensity of the fluorescence signal reaches a relatively stable tendency. But at the same time, with the increase of substrate concentration, the background signal was also enhanced. After comprehensive consideration, the concentration of DNAzyme substrate was selected as 200 nM, and the ratio of DNAzyme structure was 3:1. Subsequent experiments were carried out under these conditions.

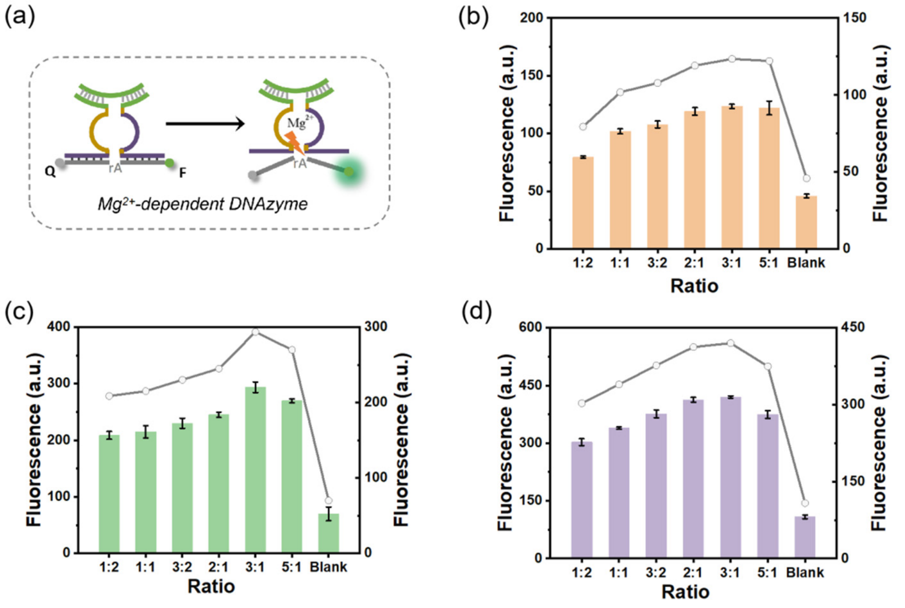

Figure S2. Optimization of structure concentration and the ratio of DNAzyme. (a) Schematic diagram of the process that DNAzyme substrate cutting and releasing fluorescent signal. The relationship between different substrate concentrations and DNAzyme structure in different proportions with fluorescence signal intensity, when substrate is 100 nM (b), 200 nM (c), and 300 nM (d).

Detection performance of DNAzyme

To explore the performance of DNAzyme detecting targets, different concentrations of DNAzyme-open were detected under the same experimental conditions. Figure S3 shows that the intensity of the fluorescence signal gradually decreases with the decrease of the DNAzyme-open concentration. When the final concentration of DNAzyme-open is 1 nM, the experimental group and the blank control can still be distinguished by the intensity of the fluorescence signal, which proves that the DNAzyme structure has the ability to detect the target sequence sensitively.

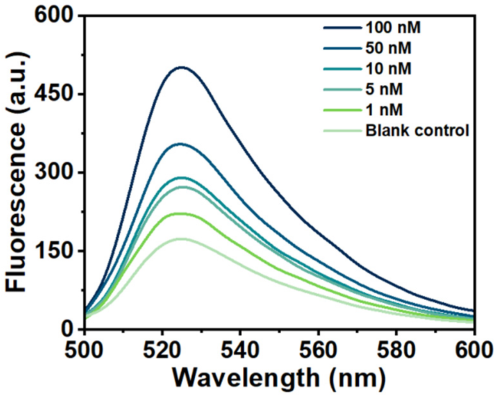

Figure S3. Fluorescence emission spectra in the addition of DNAzyme-open with different concentrations (from bottom to top): 0, 1 nM, 5 nM, 10 nM, 50 nM and 100 nM.

Subsequently, the specificity of DNAzyme structure for target detection was further explored. The intensity of the fluorescence signal can effectively distinguish the target sequence from other mutant base sequences (Figure S4), indicating that the DNAzyme structure has high specificity. The sequences used in the specifity evaluation of DNAzyme structure are shown in Table S2.

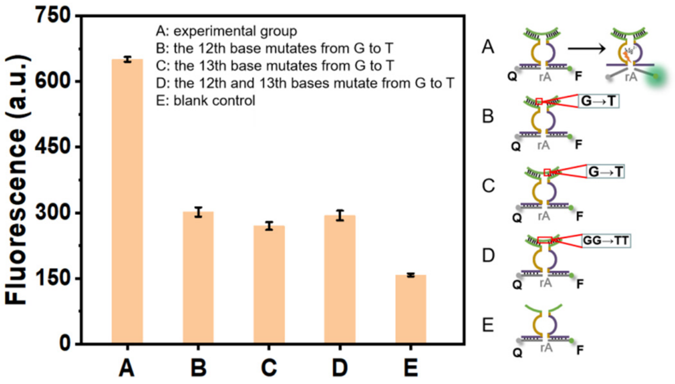

Figure S4. Specificity assessment of DNAzyme structure. Under the same experimental conditions (the final concentration of target was 100 nM), the fluorescence signals of different targets (DNAzyme-open, DNAzyme-open12, DNAzyme-open13, DNAzyme-open1213 and blank control) were detected.

Table S2. Nucleic acid sequences used in the specifity evaluation of DNAzyme structure.

Name	Sequences（5′- 3′）
DNAzyme-open12	CTACC CTCCA GTGTC AACAA GGAG (22nt)
DNAzyme-open13	CTACC CTCCA GGTTC AACAA GGAG (22nt)
DNAzyme-open1213	CTACC CTCCA GTTTC AACAA GGAG (22nt)

Note: the red bases are sequence mutation sites.

Detection of miR-21 by spike recovery

We used DEPC as a baseline and tested the fluorescence emission spectra of DEPC and blank control before the experiment. The detection results in cell supernatant and DEPC are shown in Figure S5. We also tested the spike recovery ability of RCA-DNAzyme assay on miR-21 in DEPC (Figure S6). For targets with a concentration in the dynamic range of 10 ~ 5000 pM, the recoveries were in the range from 97.40% to 106.80%. Moreover, the relative standard was lower than 5.2% (Table S3), indicating the acceptable accuracy of the sensing strategy.

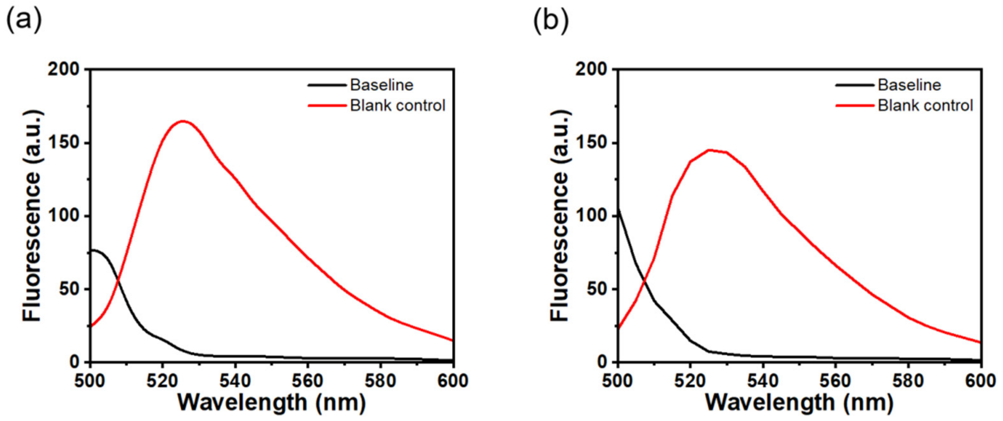

Figure S5. Fluorescence emission spectra of baseline and blank control in cell supernatant (a) and DEPC (b).

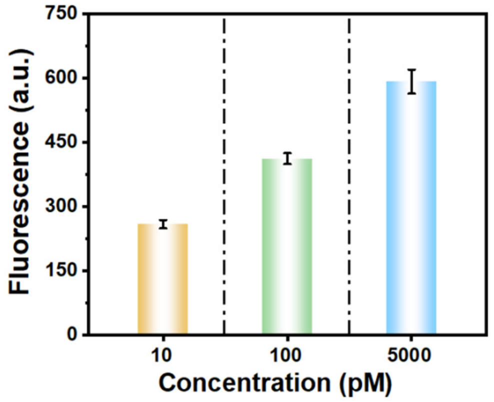

Figure S6. Fluorescence intensities of miR-21 spike recovery detection with different concentrations (10 pM, 100 pM and 5000 pM) in DEPC.

Table S3. The results of spike recovery in DEPC..

Samples	Spiked (pM)	Detected (pM)	Recovery (%)	RSD (%)
1	10	10.68	106.80	3.80
2	100	97.40	97.40	2.90
3	5000	5209.40	104.19	5.20

Relative standard deviation (RSD) was obtained by three parallel experiments.

Table S4. Comparison of different methods for RNA detection.

System	Linear range	Detection limit	Reference
DNAzyme walker	0.1 nM - 35 nM	34 pM	[1]
DNAzyme + HCR	25 pM - 30 nM	14.8 pM	[2]
CHA + FRET	500 pM - 50 nM	28.3 pM	[3]
DNAzyme	1 nM - 100 nM	650 pM	[4]
RCA + DNAzyme	1 pM - 10 nM	527 fM	This work

References

1. Liu, C.; Hu, Y.; Pan, Q.; Yi, J.; Zhang, J.; He, M.; He, M.; Chen, T.; Chu, X., A microRNA-triggered self-powered DNAzyme walker operating in living cells. *Biosens. Bioelectron.* **2019**, *136*, 31-37.
2. Zhan, J.; Liu, Z.; Liu, R.; Zhu, J.-J.; Zhang, J., Near-Infrared-Light-Mediated DNA-Logic Nanomachine for Bioorthogonal Cascade Imaging of Endogenous Interconnected MicroRNAs and Metal Ions. *Anal. Chem.* **2022**, *94*, 16622-16631.
3. Li, L. L.; Lv, W. Y.; Xu, Y. T.; Li, Y. F.; Li, C. M.; Huang, C. Z., DNA Logic Nanodevices for the Sequential Imaging of Cancer Markers through Localized Catalytic Hairpin Assembly Reaction. *Anal. Chem.* **2022**, *94*, 4399-4406.
4. Huang, X.; Zhang, Y. F.; Chen, J.; Zhang, L.; Xu, Y. Z.; Yin, W.; Shi, Y. K.; Zou, X. Y.; Liu, S. Y.; Dai, Z., Dual-Locked DNAzyme Platform for In Vitro and In Vivo Discrimination of Cancer Cells. *Anal. Chem.* **2022**, *94*, 12221-12230.
